# Deep learning-assisted ultra-fast/low-dose whole-body PET/CT imaging

**DOI:** 10.1007/s00259-020-05167-1

**Published:** 2021-01-25

**Authors:** Amirhossein Sanaat, Isaac Shiri, Hossein Arabi, Ismini Mainta, René Nkoulou, Habib Zaidi

**Affiliations:** 1 Division of Nuclear Medicine and Molecular Imaging, Geneva University Hospital, CH-1211 Geneva, Switzerland; 2 Geneva University Neurocenter, Geneva University, 1205 Geneva, Switzerland; 3 Department of Nuclear Medicine and Molecular Imaging, University of Groningen, University Medical Center Groningen, Groningen, Netherlands; 4 Department of Nuclear Medicine, University of Southern Denmark, DK-500 Odense, Denmark

**Keywords:** PET/CT, Whole-body imaging, Low-dose imaging, Deep learning, Lesion detectability

## Abstract

**Purpose:**

Tendency is to moderate the injected activity and/or reduce acquisition time in PET examinations to minimize potential radiation hazards and increase patient comfort. This work aims to assess the performance of regular full-dose (FD) synthesis from fast/low-dose (LD) whole-body (WB) PET images using deep learning techniques.

**Methods:**

Instead of using synthetic LD scans, two separate clinical WB ^18^F-Fluorodeoxyglucose (^18^F-FDG) PET/CT studies of 100 patients were acquired: one regular FD (~ 27 min) and one fast or LD (~ 3 min) consisting of 1/8^th^ of the standard acquisition time. A modified cycle-consistent generative adversarial network (CycleGAN) and residual neural network (ResNET) models, denoted as CGAN and RNET, respectively, were implemented to predict FD PET images. The quality of the predicted PET images was assessed by two nuclear medicine physicians. Moreover, the diagnostic quality of the predicted PET images was evaluated using a pass/fail scheme for lesion detectability task. Quantitative analysis using established metrics including standardized uptake value (SUV) bias was performed for the liver, left/right lung, brain, and 400 malignant lesions from the test and evaluation datasets.

**Results:**

CGAN scored 4.92 and 3.88 (out of 5) (adequate to good) for brain and neck + trunk, respectively. The average SUV bias calculated over normal tissues was 3.39 ± 0.71% and − 3.83 ± 1.25% for CGAN and RNET, respectively. Bland-Altman analysis reported the lowest SUV bias (0.01%) and 95% confidence interval of − 0.36, + 0.47 for CGAN compared with the reference FD images for malignant lesions.

**Conclusion:**

CycleGAN is able to synthesize clinical FD WB PET images from LD images with 1/8th of standard injected activity or acquisition time. The predicted FD images present almost similar performance in terms of lesion detectability, qualitative scores, and quantification bias and variance.

**Supplementary Information:**

The online version contains supplementary material available at 10.1007/s00259-020-05167-1.

## Introduction

Good image quality and high quantitative accuracy in ^18^F-Fluorodeoxyglucose (^18^F-FDG) PET imaging are crucial for reliable visual interpretation and image analysis in clinical oncology [1, 2]. Apart from the technical aspects, PET image quality depends on the amount of the injected radiotracer and/or acquisition time, which are proportional to the statistics of the detected events and hence the noise characteristics of PET images. The main argument in favor of reducing the injected radiotracer’s activity is the potential hazards of ionizing radiation [3]. Albeit low, this risk motivates precaution, particularly in pediatric patients, healthy volunteers or in case of multiple scanning for follow-up or treatment response monitoring using different molecular imaging probes. Reducing the acquisition time positively impacts patients’ comfort and increases PET scanner throughput. However, dose/time reduction adversely affects image quality, hence potentially reducing signal-to-noise ratio (SNR) and hampering the diagnostic and quantitative performance of PET imaging.

During recent years, deep learning algorithms were deployed for various medical image analysis tasks, exhibiting superior performance over traditional strategies [4–10]. Conventional post-reconstruction PET denoising approaches, such as Gaussian, bilateral and non-local mean filtering, are commonly used in clinical and research settings. However, they could also induce noticeable signal loss [11, 12], in addition to difficulties in setting the hyperparameters to achieve the desirable output owing to variable noise characteristics in PET images [13, 14]. Contrary to conventional denoising approaches which operate directly on low-dose (LD) PET images, deep learning algorithms are capable of learning a non-linear transformation to predict full-dose (FD) from LD images [15].

Several recent studies have shown the potential of LD to FD conversion in various body regions (e.g., brain, chest, abdomen, and pelvis). For example, a recent study performed by Chen et al. utilized 2D slices of LD ^18^F-Florbetaben brain PET images along with various MR sequences to predict FD images using a U-Net architecture [16]. More recently, Sanaat et al. suggested a deep learning algorithm for training the data in projection space instead of image space to synthesize FD brain sinograms from corresponding LD sinograms [17]. Lu et al. showed that a 3D UNET model trained with only 8 LD images of lung cancer patients generated with 10% of the corresponding FD images effectively reduced the noise while minimizing the bias in lung nodules [18]. Gong et al. proposed a deep neural network (DNN) for denoising brain and lung ^18^F-FDG PET images [19]. Labeled images were generated for training by summation of an hour-long dynamic PET scan into a FD frame whereas the LD images were obtained by decimating the FD scan to 1/5^th^ of the counts. Kaplan et al. trained a residual convolutional neural network (CNN) to estimate FD images from 1/10^th^ of the counts of FD scans separately for various body regions (brain, chest, abdomen, and pelvis) using a single study with testing performed on another study [20]. More recently, Zhou et al. proposed a supervised deep learning model using a small dataset consisting of 18 thoracic PET images to synthesize FD from LD scans [21].

Few studies with different degrees of success investigated the potential of LD to FD image conversion in whole-body (WB) PET imaging. Almost all of them suffer from small sample size used for training and lack of comprehensive clinical assessment. Cui et al. presented an unsupervised denoising model that does not require paired datasets for training, where the model was fed by the patient’s prior FD and current LD PET images to predict a high quality PET image [22]. Lei et al. proposed a cycle-consistent generative adversarial network (CycleGAN) model to predict FD from 1/8^th^ of FD WB ^18^F-FDG PET images [23]. The proposed model was trained and tested on 25 and 10 WB PET images, respectively. In another study, Lei et al. used a similar approach incorporating CT images into the network to aid the process of PET image synthesis from LD on a small dataset consisting of 16 patients [24].

The major concerns regarding previous WB PET studies focusing on synthesizing FD images from LD images can be grouped around three subthemes: (i) all studies included only a small sample size which decreases robustness and impacts generalization of the models, particularly to abnormal cases. (ii) Except the above referenced three studies, in all previous works, the model was trained for different body regions separately not as a single WB image. (iii) Lack of all-inclusive clinical evaluation including assessment of image quality and lesion detectability. In the present work, we compare two well-established CNN architectures, namely the residual network (ResNET) and CycleGAN models, used to predict FD from LD ^18^F-FDG WB PET images using a realistic clinical database acquired at two separate sessions with different scan durations mimicking FD and LD images (ratio of 1/8^th^). Quantitative image quality assessment and qualitative evaluation of the predicted FD from LD images were performed. To the best of our knowledge, this is the first study focusing on lesion detectability when assessing these approaches.

## Materials and methods

### PET/CT data acquisition

This prospective single-institution study was approved by the institutional ethics committee of Geneva University Hospital and all patients gave written informed consent. We included 100 consecutive patients referred to the Nuclear Medicine department for WB ^18^F-FDG PET/CT studies between May and September 2017. Fifteen studies were excluded because of technical or logistic issues (artifacts, misregistration, the difference in scanning sequences, poor quality of either LD or FD PET images). In addition, images presenting with noticeable motion artifacts and differences in time-activity curves were excluded. PET/CT scans were performed on a Biograph mCT PET/CT scanner (Siemens Healthcare, Erlangen, Germany). A low-dose CT scan (120 kVp, 80 mAs) was performed for PET attenuation correction. This was followed by a WB PET LD/fast scan (as there is a direct link between fast and LD scanning) acquired 60 min post-injection of 240 ± 50 MBq of ^18^F-FDG in continuous bed motion mode with a speed of 5 mm/s(~ 25 s/bed position). Subsequently, during the same acquisition session, a FD/standard duration scan with a speed of 0.7 mm/s (~ 3 min/bed position) was performed. Both FD and LD PET images were reconstructed using an ordinary Poisson ordered subsets-expectation maximization (OP-OSEM) algorithm (2 iterations, 21 subsets, post-reconstruction Gaussian filtering with 2 mm FWHM).

### Deep learning algorithms

**Fig. 1:**
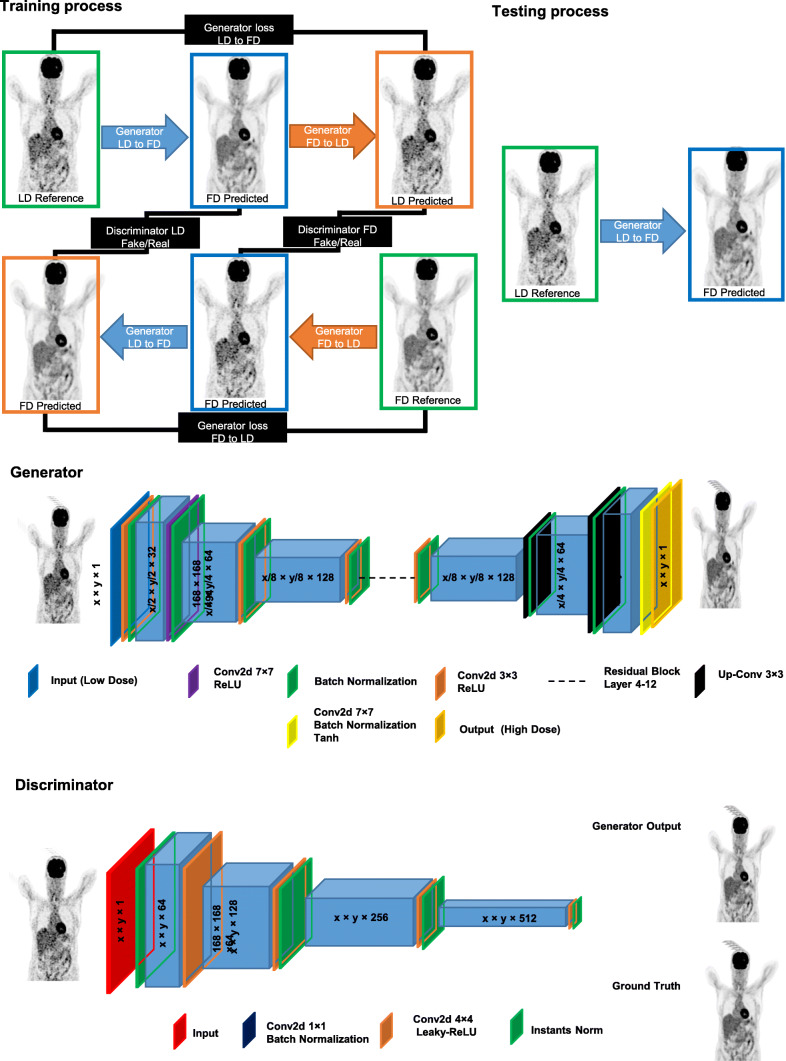
Schematic architecture of the cycle-consistent generative adversarial network (CycleGAN) model used for FD PET synthesis. The left panel depicts the training process whereas the right panel shows the process of testing and the structure of the generator and discriminator

We used two state-of-the-art deep learning algorithms, namely ResNET and CycleGAN models. The details of the deep learning approaches and architectures of the networks are presented in the “Supplementary information” section. The flowchart of CycleGAN architecture is presented in Fig. 1. The motivation behind the choice of these architectures instead of popular networks, such as UNET, is that the adopted CycleGAN internally uses a UNET-like structure (generator based on UNET). In addition, although ResNET is a non-standard network for image synthesis, our trial and error experiments revealed that it outperforms UNET. The training and hyperparameter tuning of the models were performed on 60 patients. Ten patients were used for model evaluation whereas a separate unseen dataset of 15 patients served as a test (external validation) dataset. For data normalization, we converted PET images to standardized uptake values (SUVs) and then divided them by a SUV_max_ of 10.

The deep learning models were implemented on NVIDIA 2080Ti GPU with 11 GB memory running under the windows 10 operating system. The training was performed using a mini-batch size of 6 for 215 epochs. We opted for not using cross-validation since recent guidelines seem to suggest that although multiple internal cross-validation can be useful, independent validation using an external dataset for a single trained model is preferred over internal validation to properly evaluate generalizability [25, 26].

### Evaluation strategy

#### Clinical qualitative assessment

ET images predicted with ResNET and CycleGAN models (denoted as RNET and CGAN, respectively) along with their corresponding reference FD and LD PET images were anonymized and randomly enumerated for qualitative evaluation by two experienced nuclear medicine physicians (this process was done for each patient). In total, 100 PET images were evaluated, including 25 reference FD, 25 LD, 25 RNET, and 25 CGAN PET images belonging to the test and validation datasets. The quality of PET images was assessed in three steps. First, the two physicians, with over 15 years of experience, were asked to use a 5-point grading scheme for visual image quality assessment, namely (1) uninterpretable, (2) poor, (3) adequate, (4) good, and (5) excellent. In the second step, since image quality does not guarantee lesion detectability in clinical practice, the two physicians were asked to express their overall assessment of the diagnostic quality of PET images with a binary decision (accepted or failed). Lastly, the physicians assessed lesion detectability and drew regions-of-interest (ROIs) on malignant lesions. The size of ROIs was defined to include the whole lesion. This process was performed separately for the brain and neck + trunk regions. The region-wise performance of the model was performed to mitigate potential bias between regions with high and low count statistics.

#### Quantitative analysis

The accuracy of the predicted FD from LD PET images was evaluated using three quantitative metrics, including the mean squared error (MSE), peak signal-to-noise ratio (PSNR), and structural similarity index metrics (SSIM) (Eqs. 1–3). Moreover, these metrics were also calculated for the LD images to provide an insight about the noise levels and significant signal loss.

1 $$ \mathrm{MSE}\left(R,P\right)=\frac{\sum_{j=1}^T{\left({R}_j-{P}_j\right)}^2}{T} $$

2 $$ \mathrm{PSNR}\left(R,P\right)=20\times {\mathit{\log}}_{10}^{\left(\frac{\operatorname{Max}(R)}{\sqrt{\mathrm{MSE}\left(R,P\right)}}\right)}\kern0.5em $$

3 $$ \mathrm{SSIM}\left(R,P\right)=\frac{\left(2{m}_R{m}_P+{c}_1\right)\left(2{\sigma}_{RP}+{c}_2\right)}{\left({m}_R^2+{m}_P^2+{c}_1\right)\left({\sigma}_R^2+{\sigma}_P^2+{c}_2\right)}\kern0.5em $$

In Eq. (1), *T* is the total number of voxels, *R* is the reference image (FD), and *P* is the predicted image. In Eq. (2) Max(*P*) indicates the maximum intensity value of *R* or *P*, whereas MSE is the mean squared error. *m*_*r*_ and *m*_*p*_ in Eq. (3) denote the mean value of the images *R* and *P*, respectively. *σ*_*RP*_ indicates the covariance of *R* and *P*. ${\sigma}_R^2\  and\ {\sigma}_P^2$ in turn represent the variances of *R* and *P* images, respectively. The constant parameters *c*_1_ and *c*_2_ (*c*_1_ = 0.01 and *c*_2_ = 0.02) were used to avoid a division by very small numbers.

Region-based analysis was also performed to assess the agreement in tracer uptake between predicted and FD images. Using the AMIDE software [27], 4 ROIs were manually drawn over the liver, brain, and left/right sides of the lungs. Given the ROIs, the region-wise SUV bias and standard deviation (STD) were calculated for each region on the predicted FD and LD PET images considering the FD PET images as standard of reference. Bland-Altman plots of SUVs in the four ROIs delineated on normal organs and the 285 malignant lesions were calculated (82, 92, and 111 lesions were depicted on LD, RNET, and CGAN, respectively). Moreover, a joint histogram analysis was also carried out to depict the voxel-wise correlation of the tracer uptake between RNET/CGAN and reference FD PET images.

The MedCalc software [28] was employed for the pairwise comparison of MSE, SSIM, and PSNR between LD, CGAN, RNET, and reference FD PET images using paired *t* test. Bonferroni correction for multiple comparisons was applied and the significance level was set at a *P* value < 0.025 for all comparisons. The agreement between and within the physicians’ scoring was assessed using weighted Cohen’s Kappa and Krippendorff alpha was calculated to evaluate inter-rater reliability of image quality scores and ^18^F-FDG uptake patterns analysis.

## Results

We included malignant disease-free patients (patients with inflammatory or suspected infectious diseases (5.9%)) and patients with various oncological indications, including lymphoma (23.5%), lung cancer (21.2%), breast cancer (15.3%), head and neck cancer (7.1%), colorectal cancer (3.5%), and other malignancies (23.5%) (Table 1).

**Table 1:** Demographics of patients included in this study

	Training	Test	Validation
Number	60	15	10
Injected activity (MBq)	240 ± 50	235 ± 40	235 ± 47
Male/female	36/24	9/6	5/5
Age (mean ± SD)	58 ± 3	63 ± 12	71 ± 7
Weight (mean ± SD)	71 ± 8	59 ± 11	67 ± 9
Indication/diagnosis	Oncological studies include lymphoma (23.5%), lung (21.2%), breast (15.3%), head and neck (7.1%), colorectal (3.5%), other (23.5%), non-oncologic scans (5.9%)		

## Assessment of image quality

**Fig. 2:**
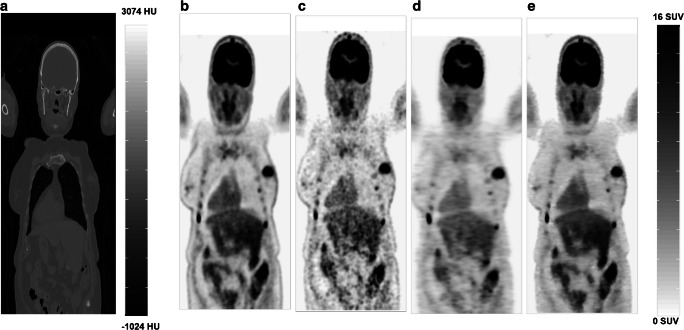
Representative ^18^F-FDG WB PET image of a 66-year old female patient. **a** Low-dose CT images used for attenuation correction, **b** reference FD images, **c** the corresponding LD images, and the predicted FD images using **d** ResNet (RNET) and **e** CycleGAN (CGAN)

PET images predicted by both deep learning models (RNET and CGAN) exhibited notable enhancement in image quality compared to LD by providing almost similar visual appearance with respect to reference FD PET images. The visual inspection revealed that the images derived by CGAN better reflected the underlying ^18^F-FDG uptake patterns and anatomy than those predicted by the RNET model (Fig. 2). Since the test and validation datasets had approximately similar RMSE, SSIM, and PSNR and similar trend with respect to clinical assessment, the results were merged and presented in a single figure. PET images predicted by CGAN showed the highest PSNR, SSIM, MSE, better noise properties, and higher quantitative accuracy with statistically significant differences with respect to RNET (Tables 2 and 3).

**Table 2:** Comparison of the results obtained from analysis of image quality of LD PET images and images predicted using ResNet (RNET) and CycleGAN (CGAN) for the test and validation datasets. SSIM, structural similarity index metrics; PSNR, peak signal to noise ratio; RMSE, root mean squared error

	MSE	SSIM	PSNR
Validation dataset			
CGAN	0.03 ± 0.04	0.98 ± 0.08	41.08 ± 3.90
RNET	0.12 ± 0.10	0.94 ± 0.10	35.41 ± 5.56
LD	0.15 ± 0.09	0.89 ± 0.11	31.21 ± 3.08
*P* value (CGAN vs. RNET)	0.012	0.018	0.035
*P* value (CGAN vs. LD)	0.022	0.011	0.017
*P* value (RNET vs. LD)	0.051	0.025	0.041
Test dataset			
CGAN	0.03 ± 0.07	0.97 ± 0.02	39.08 ± 3.56
RNET	0.13 ± 0.10	0.93 ± 0.04	34.91 ± 1.50
LD	0.17 ± 0.04	0.9 ± 0.03	29.21 ± 2.43
*P* value (CGAN vs. RNET)	0.021	0.015	0.015
*P* value (CGAN vs. LD)	0.013	0.03	0.022
*P* value (RNET vs. LD)	0.021	0.011	0.021

**Table 3:** SUV bias, average and absolute average of SUV bias ± STD calculated across the four standard non-lesional tissue areas and 100 malignant lesions for LD, RNET, and CGAN PET images

SUV bias	LD	RNET	CGAN
SUV bias in left lung (%)	4.83 ± 3.25	− 3.22 ± 2.12	4.23 ± 1.3
SUV bias in right lung (%)	1.34 ± 4.11	− 2.92 ± 2.22	3.82 ± 0.88
SUV bias in liver (%)	4.54 ± 1.32	− 3.20 ± 4.23	2.32 ± 2.50
SUV bias in brain (%)	-8.40 ± 6.2	− 6.01 ± 3.65	3.21 ± 4.17
Average SUV bias for all 4 regions (%)	0.57 ± 5.36	− 3.83 ± 1.25	3.39 ± 0.71
Absolute average SUV bias for all 4 regions (%)	4.78 ± 2.49	3.83 ± 1.25	3.39 ± 0.71
Average SUV bias in malignant lesions (%)	6.00 ± 1.97	− 9.42 ± 7.20	2.03 ± 7.60
Absolute average SUV bias in malignant lesions (%)	14.30 ± 2.13	11.86 ± 6.20	9.24 ± 1.01
*P* value (CGAN vs. RNET)	< 0.02	< 0.01	< 0.02
*P* value (CGAN vs. LD)	< 0.02	< 0.05	< 0.02
*P* value (RNET vs. LD)	< 0.05	< 0.05	< 0.05

## Clinical readings

Weighted Cohen’s Kappa and Krippendorff alpha tests were used to evaluate between and within raters’ agreements. Inter- and intra-reader agreement of image quality scores and ^18^F-FDG uptake patterns analysis were high (Krippendorff alpha for all comparisons was > 8) while the Kappa was more than 0.7 for the failed/ accept task, except for LD where it was equal to 0.52.

**Fig. 3:**
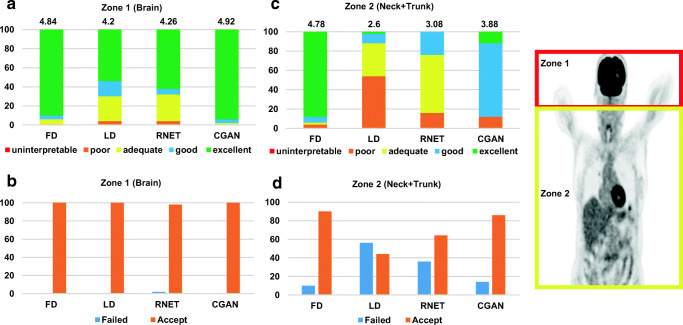
Top panel: image quality assessment by the two nuclear medicine physicians for LD, FD, RNET and CGAN PET images. Mean scores are presented on the top of the bar plots. 1 = uninterpretable, 2 = poor, 3 = adequate, 4 = good, 5 = excellent. Bottom panel: Percentage of failed and accepted images is illustrated. Failed was assigned whenever lesion detectability was not good compared to the image displaying best image quality. The two defined anatomical regions are shown on the right

The quality of LD images was poor (score = 2.6) with the highest percentage of failed cases (56%) for neck and trunk region while achieving relatively good quality (score = 4.2) with zero failed case in the brain region (Fig. 3). CGAN outperformed RNET by synthesizing images with near good quality (score = 3.88) and 86% accepted cases for the neck and trunk region while achieving near excellent quality (score = 4.92) and 100% accepted cases for the brain region.

**Fig. 4:**
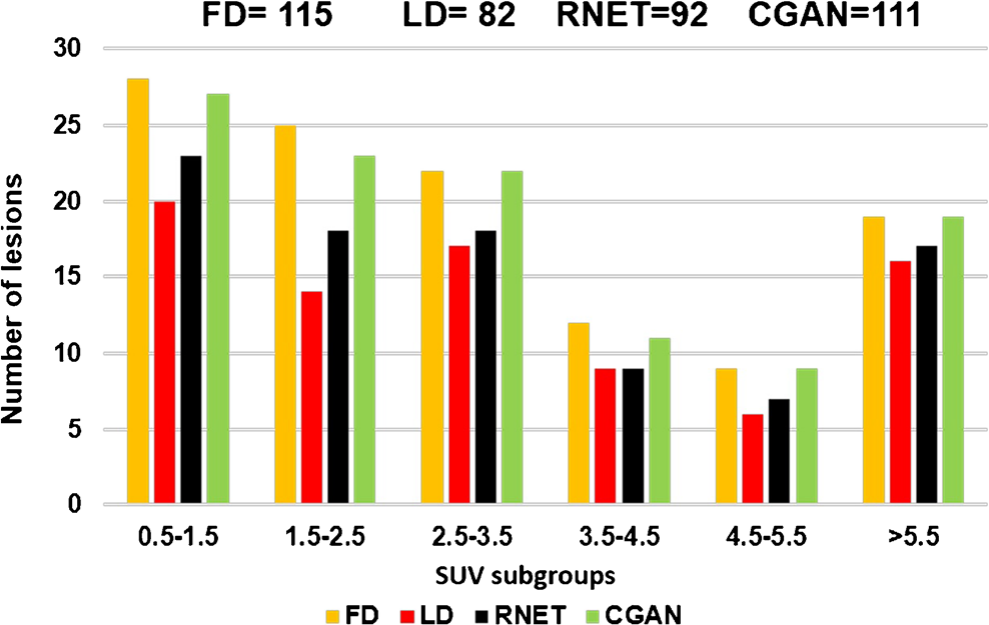
Lesion detectability histogram according to different SUV subgroups for FD, LD, RNET, and CGAN evaluated by two expert nuclear medicine physicians

With regard to lesion detectability, both CGAN and RNET performed well (depicting 19 and 17 out of 19 lesions, respectively) for lesions with high uptake (SUV > 5.5). However, CGAN performed much better compared to RNET (depicting 27 and 23 out of 28 lesions, respectively) for lesions with reduced SUV (0.5 < SUV < 1.5) and smaller size (Fig. 4). It should be noted that all detected lesions pinpointed by physicians on FD images were also identified on LD, CGAN, and RNET.

**Fig. 5:**
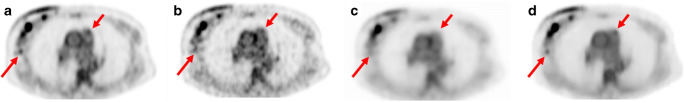
Representative example of lesion detectability showing a clinical study with multifocal multicentric breast cancer with skin involvement of the right breast and extensive lymph node metastases including the mediastinal nodes comparing FD and LD images as well as the synthesized images using both networks. The lesion in the infero-extrenal quadrant (long red arrow) was detected on **a** FD, **b** LD, and **d** CGAN, but missed on RNET (**c**). The mediastinal lymph node (level 6) (short red arrow) was visible on FD and CGAN and missed on LD and RNET

Figure 5 illustrates a representative example of multifocal multicentric breast cancer with skin involvement of the right breast and extensive lymph node metastases including mediastinal nodes comparing FD and LD images as well as the synthesized images using both networks. It can be seen that some lesions and lymph nodes are visible on HD and CGAN, but missed by LD and RNET.

## Region-based analysis

**Fig. 6:**
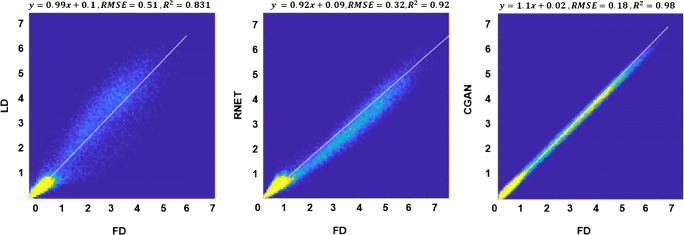
Joint voxel-wise SUV histogram analysis of the LD PET images (left), predicted FD images using ResNet (middle), and CycleGAN (right) versus FD PET images. For better illustration, the plot was limited to maximum SUV of 7

Linear regression plots depicting the correlation between tracer uptake for LD, RNET, and CGAN with respect to FD are shown in Fig. 6. The scatter and linear regression plots showed higher correlation between CGAN and FD (*R*^2^ = 0.98, RMSE = 0.18) compared to RNET (*R*^2^ = 0.92, RMSE = 0.32). A relatively higher RMSE (0.51) was obtained for LD PET images. All pair-wise *t* tests (accounting for three comparisons) had *P* values < 0.001.

**Fig. 7:**
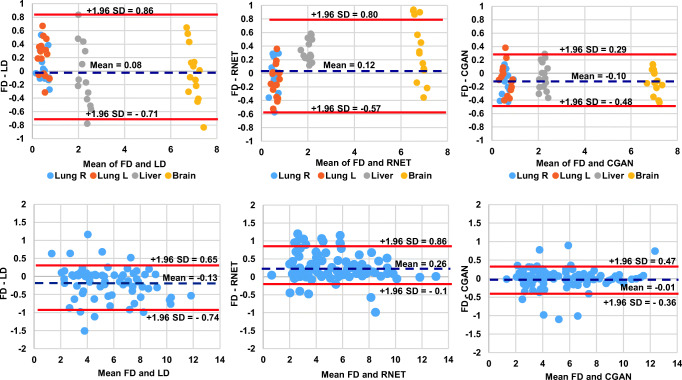
The top row shows the Bland-Altman plots of SUV_mean_ differences in the 4 normal organ regions. Second row shows the SUV_max_ for the malignant lesions calculated for LD (left), RNET (middle), and CGAN (right) PET images with respect to reference FD PET images in the test dataset. The solid red and dashed blue lines denote the mean and 95% confidence interval (CI) of the SUV differences, respectively

The Bland-Altman plots showed that the lowest SUV bias (− 0.10, − 0.01) and the smallest SUV variance (95% CI: − 0.48, + 0.29, 95% CI: − 0.36, + 0.47) were achieved by CGAN for normal organs and malignant lesions, respectively. Though the SUV bias is extremely low for LD images, increased variance compared with FD images was observed (95% CI: − 0.71, + 0.86 for normal organs and 95% CI: − 0.74, + 0.65 for malignant lesions), reflecting poor image quality and high noise characteristics (Fig. 7).

The SUV bias was below 8.4% for CGAN, RNET, and LD images with LD images exhibiting a relatively higher standard deviation compared to RNET and CGAN. CGAN led to the lowest absolute average SUV bias (3.39 ± 0.71%) across all four organs, while RNET and LD resulted in SUV bias of 3.83 ± 1.25% and 4.78 ± 2.49%, respectively. Even though a low SUV bias was observed in LD images, a remarkably higher standard deviation was obtained, reflecting the high noise characteristics in LD images (Tables 3 and 4).

**Table 4:** Standard deviation of SUV for each ROI drawn in each organ for LD, RNET, and CGAN PET images

	FD	LD	RNET	CGAN
Left lung	0.17	0.74	0.38	0.22
Right lung	0.11	0.89	0.29	0.16
Liver	0.53	1.34	0.83	0.52
Brain	0.71	1.21	0.91	0.62

## Discussion

The main aim of the present study was to generate diagnostic quality WB ^18^F-FDG PET images from LD PET images corresponding to 1/8^th^ of standard FD acquisition time. In contrast to previous studies, we aimed at training the network with realistic images obtained from two separate scans acquired with standard injected activity but different continuous motion bed speeds and to evaluate the performance of the two DNN algorithms for estimation of FD PET images. It was shown that CGAN had a superior image quality and lower regional SUV bias and variance compared to RNET. This highlights that CGAN adds more constraints to the generator by introducing an inverse transformation in a circular manner, which more effectively avoids model collapse and better ensures that the generator finds a unique meaningful mapping. Our current study demonstrated the improved performance of CGAN over RNET for addressing the PET image denoising problem, particularly in terms of lesion detectability.

The assessment of image quality performed by nuclear medicine physicians demonstrated the superior performance of the CGAN approach, showing close agreement between CGAN and reference FD images. The achieved MSE was 0.03 ± 0.04, 0.12 ± 0.10, and 0.15 ± 0.09 for LD and synthesized CGAN and RNET images, respectively, reflecting the effectiveness of CGAN model (*P* value < 0.05). Moreover, the SSIM improved from 0.89 ± 0.11 for LD images to 0.94 ± 0.10 for RNET and further to 0.98 ± 0.08 for CGAN. It would be enlightening to consider the resulting metrics in conjunction with those obtained from LD images for better interpretation of the extent of improvement achieved by the proposed methods.

Lei et al. [29] reported that WB LD images created from 1/8^th^ of the equivalent FD images achieved an average mean error (ME) and normalized mean square error (NMSE) of − 0.14 ± 1.43% and 0.52 ± 0.19% using a CGAN model while the LD PET images achieved a ME of 5.59 ± 2.11% and NMSE of 3.51 ± 4.14%. The normalized cross-correlation (NCC) was improved from 0.970 to 0.996, while the PSNR increased from 39.4 to 46.0 dB using the CGAN model with respect to LD images. The NCC metric reflects mainly the correlation between two signals (or images) in terms of pattern and/or texture while it is less sensitive to the intensity of signals. Conversely, the SSIM metric, which measures the perceptual difference between two signals, reflects the quantitative accordance between two images. Since the above mentioned study did not report the SSIM, we calculated the NCC for LD (0.87), RNET (0.93), and CGAN (0.97) for comparison with our results. In another study performed by the same group, CT images along with LD PET images were fed to a CGAN to achieve significant improvement of the ME (< 1%) for synthesized FD compared to corresponding LD images (5.59%) [24].

For clinical evaluation, WB PET images were split into brain and neck + trunk regions. The motivation behind is the intense ^18^F-FDG uptake in the brain, which is considerably higher than other biologically normal tissues and organs in the body. Hence, by reducing the injected activity or acquisition time, the level and/or properties of the induced noise in the brain region would differ from those in the chest/abdomen. Another reason guiding our choice to group neck and torso in our assessment was to facilitate restaging using fast and standard acquisitions. This assessment revealed that the qualitative scores assigned to the predicted images in the brain region (4.92/5) were significantly higher than those assigned to the chest/abdomen (3.88/5) when using CGAN.

We included patients with various conditions, such as age, weight and height, and cancer type for training, evaluation, and independent validation datasets, to provide a heterogeneous sample reflecting common clinical practice. The Bland and Altman analysis showed lower SUV bias and variance in the 4 organs and 285 malignant lesions when using CGAN and RNET compared to LD images. The results further demonstrated the superior performance of the CGAN approach, resulting in SUV values comparable to those produced by the original FD images. In terms of computational time, the training of ResNET is less demanding (~ 40 h) than for CycleGAN network (~ 95 h). Moreover, the synthesis of a 3D PET image (after training) using ResNET takes ~ 80 s versus ~ 250 s required by CycleGAN.

It should be emphasized that, in this work, the LD images were obtained through a separate fast PET acquisition corresponding to ~ 1/8^th^ the FD scan duration prior to FD PET acquisition. However, most related works in the literature employed a random sampling scheme from the recorded events (in listmode format) of the standard FD PET acquisition to generate the LD PET images. There are a number of fundamental differences between LD images generated through decimating the FD scan and LD images actually acquired separately by reducing the acquisition time or the injected activity. First, when the LD PET image is obtained from a separate acquisition, the underlying PET signal may be different between LD and FD PET images owing to the varying tracer kinetics of the radiotracer during the course of imaging. Moreover, potential patient motion between these two scans further adds to the complexity of FD PET estimation from the fast/LD PET scan. This bias was minimized by first acquiring the LD PET images and comfortable positioning of patients. Second, since fast PET acquisition is performed while the activity concentration in the field-of-view is equal to that of a standard PET scan, the number of recorded random events, which increases quadratically with the injected dose, is higher. As such, the fast scan would contain higher noise level compared to the equivalent real or simulated (decimated) LD scan [30].

The current study inherently bears a number of limitations. First, during the clinical evaluation, the LD images were relatively easy to identify by physicians. Hence, they could have been subconsciously biased and intuitively assigned lower scores to these images. The acceptance or failure of an image with respect to the clinical information it carries was essentially based on lesion detectability criteria, with the brain obtaining a higher rate of acceptable cases than corresponding neck and trunk images. This discrepancy may be due to the fact that most patients, except two, did not have lesions in the brain. Moreover, patient motion during the two PET/CT scans, particularly for elderly patients, might impair the image quality of both LD and FD PET images. In addition, the evaluation process was performed using only ^18^F-FDG as radiotracer and a single PET/CT scanner model. Different radiotracer distribution and concentration as well as other PET/CT devices and multicentric images would need reappraisal using our training networks. In this regard, the concept of transfer learning can be used for retraining images acquired with other radiotracers and PET scanners, which might help mitigating the limited size of training datasets.

## Conclusion

We have demonstrated that high-quality WB ^18^FDG PET images can be generated using deep learning approaches. The noise was effectively reduced in the predicted FD PET images from the LD images. An important finding of this work is that the use of quantitative metrics is not sufficient to evaluate model performance. The clinical evaluation indicated that models (e.g., ResNET) achieving relatively good quantitative performance do not perform well when considering clinical tasks. The prediction of FD PET images using CycleGAN model exhibited superior performance, resulting in higher image quality, minimal quantification bias, and closer lesion detectability performance relative to the standard of reference.

## Supplementary Information

ESM 1 (PDF 721 kb)
