# Supplementary material for: Deep learning-assisted ultra-fast/low-dose whole-body PET/CT imaging
Source: Eur J Nucl Med Mol Imaging. 2021 Jan 25;48(8):2405–15. doi: 10.1007/s00259-020-05167-1 (PMC8241799; doi:10.1007/s00259-020-05167-1)
Supplement: Supplementary file 1 — (PDF 721 kb) [file 259_2020_5167_MOESM1_ESM.pdf]

SUPPLEMENTARY TEXT

ResNET Details

ResNET (1) is composed of 20 convolutional layers where different levels of feature extraction performed with dilation concept, a 3×3×1 convolution kernel with zero dilation for the first seven layers (for low-level features extraction), the next seven layers with a dilation of two (for medium-level features extraction) and the last six layer with a dilation by a factor of four (for high level feature extraction). Every two convolutional layers linked with residual connection and rectified linear unit (ReLU) was used as activation function. The LD^{18}F-FDG PET image was fed to Res-NET network to predict FD image in image-to-image translation approaches. The network architecture was illustrated in Supplemental figure 1.

ResNET performs image-to-image translation via dilated convolution layers, which learned a function to map the LD images to FD images. The image-to-image translation problem can be defined as a task of translating one possible representation of a scene into another. Specifically, ResNET (as elaborated in Supplemental figure 1) consists of three main parts, including a 3-level dilated convolution layers with residual connection. The LD images were used as input to the network. which then attempts to generate the FD images. The key feature of ResNET, in addition to dilated convolution kernels, is the residual connection, which bypasses the parameterized layers. The ResNet architecture benefits from 9 residual blocks, which result in a large number of receptive fields (which improves the process of feature extraction). To avoid a large number of trainable parameters, a kernel of 3×3×1 was chosen for all convolution kernels. Instead of a down-sampling/up-sampling pipeline, the ResNET architecture adopts dilated convolutions with factors of 2 and 4 for seven intermediate and six last layers to ensure effective feature extraction (Supplemental figure 1).

Implementation details

The training for FD prediction was performed using 60 pairs of LD and FD images as input/output, respectively. The ResNET model with a 2D spatial window equal to 160 ×160 pixels and batch size of 20 were used. The following setting was used for the training of the two networks: learning rate = 0.001, sample per volume = 1, optimizer = Adam, loss function = L2norm and decay = 0.0001. The optimization of the network was carried out based on the L2 loss function.

CycleGAN details

The CycleGAN consists of two main parts, namely a generator and discriminator, wherein the generator learns a mapping from the source image to the target image. Since the source and target images share a similar structure, the residual network architecture was used to implement the generators to learn based on the residual image, i.e. the difference between the source and target image, rather than all images. Discriminator networks were introduced to evaluate the synthesized image. Typically, the generator’s objective is to increase the judgement error of the discriminative network, that is to fool the discriminator

network by producing synthetic or cycle images that are indistinguishable to the input training images. The discriminators' training objective is to decrease its judgement error and enhance the ability to differentiate true from synthetic images. The generator attempts to fool the discriminator by generating images that are indistinguishable from the ground truth images. The discriminator is then trained to discriminate between the generator's output and ground truth images. The generator and discriminator networks were trained in tandem in an adversarial manner. In this work, the number of convolution layers in the discriminator architecture is 9, where 8 layers are followed by batch normalization, and the last layer is followed by a sigmoid function. The flowchart of this architecture is presented in supplemental Table 1.

CycleGAN is an open github repository (2) containing the original implementation in PyTorch (3). Several other implementations are referenced in the repository. In this work, the CycleGAN model is implemented from previous code based on Keras (4, 5) with a Tensorflow (6) backend. Supplemental Table 1 shows the network architecture and details of the layers, respectively. The last layer of the generator architecture contains a Rectified linear unit (ReLU) activation function, but the corresponding code uses tanh instead and is therefore used in this work. The suggested model settings are:

Batch size = 1, Learning rate = $2 * 10{-}4$, Linear decay from initial value to zero over the last 100 epochs, lambda = 10.0, beta_1 = 0.5, for the Adam optimizer, beta_2 = 0.999, for the Adam optimizer, loss function of discriminator and generator= Mean Absolute Error, L1 Loss.

For hyperparameter tuning we used manual search, we selected some different model hyperparameters based on our previous judgment/experience. Then the model was trained and its accuracy evaluated and the process started again. This loop is repeated until a satisfactory accuracy is obtained.

REFERENCES

1. Li W, Wang G, Fidon L, Ourselin S, Cardoso MJ, Vercauteren T, editors. On the compactness, efficiency, and representation of 3D convolutional networks: brain parcellation as a pretext task. International Conference on Information Processing in Medical Imaging; 2017: Springer.
2. Zhu J-Y, Park T, Isola P, Efros AA, editors. Unpaired image-to-image translation using cycle-consistent adversarial networks. Proceedings of the IEEE international conference on computer vision; 2017.
3. Paszke A, Gross S, Chintala S, Chanan G, Yang E, DeVito Z, et al. and A. Lerer. Automatic differentiation in pytorch. 2017.
4. Chollet F. Keras: The python deep learning library. Astrophysics Source Code Library. 2018.
5. Welander P, Karlsson S, Eklund A. Generative adversarial networks for image-to-image translation on multi-contrast MR images-A comparison of CycleGAN and UNIT. arXiv preprint arXiv:180607777. 2018.
6. Abadi M, Barham P, Chen J, Chen Z, Davis A, Dean J, et al., editors. Tensorflow: A system for large-scale machine learning. 12th {USENIX} Symposium on Operating Systems Design and Implementation ({OSDI} 16); 2016.

Supplemental Table 1. Parameters of the original CycleGAN model.

Layer	Generators
1	Convolutional-(Filters-32, Kernel size-7, Stride-1), Batch Norm, ReLU
2	Convolutional-(Filters-64, Kernel size-3, Stride-2) ,Batch Norm, ReLU
3	Convolutional-(Filters-128, Kernel size-3, Stride-2), Batch Norm, ReLU
4-12	Residual block-(Filters-128, Kernel size-3, Stride-1), Batch Norm, ReLU
13	Convolutional-(Filters-64, Kernel size-3, Stride-0.5), Fractionally strided, Batch Norm, ReLU
14	Convolutional-(Filters-32, Kernel size-3, Stride-0.5), Fractionally strided, Batch Norm, ReLU
15	Convolutional-(Filters-3, Kernel size-7, Stride-1), Batch Norm, Tanh
**Layer**	**Discriminators**
1	Convolutional-(Filters-64, Kernel size-4, Stride-2), LeakyReLU with slope 0.2
2	Convolutional-(Filters-128, Kernel size-4, Stride-2), Instance Norm, LeakyReLU with slope 0.2
3	Convolutional-(Filters-256, Kernel size-4, Stride-2), Instance Norm, LeakyReLU with slope 0.2
4	Convolutional-(Filters-512, Kernel size-4, Stride-2), Instance Norm, LeakyReLU with slop 0.2

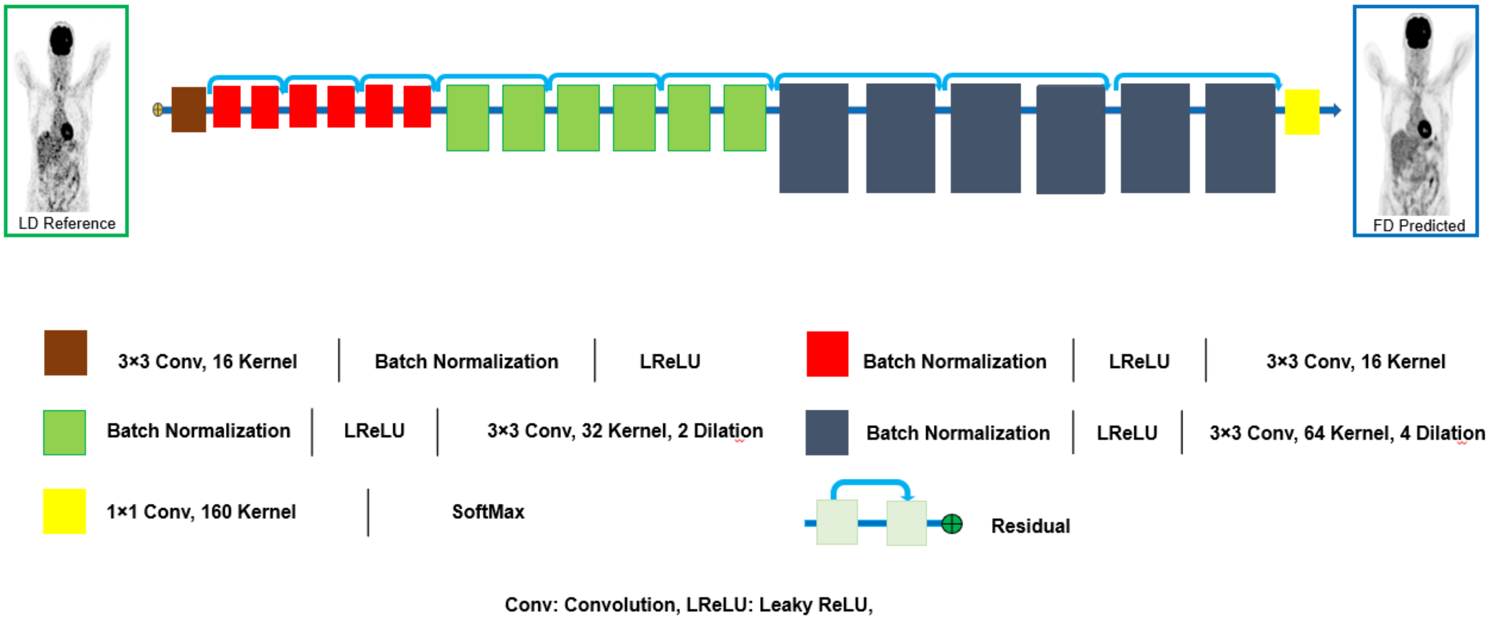

Supplemental figure 1. Schematic architecture of the Residual network.

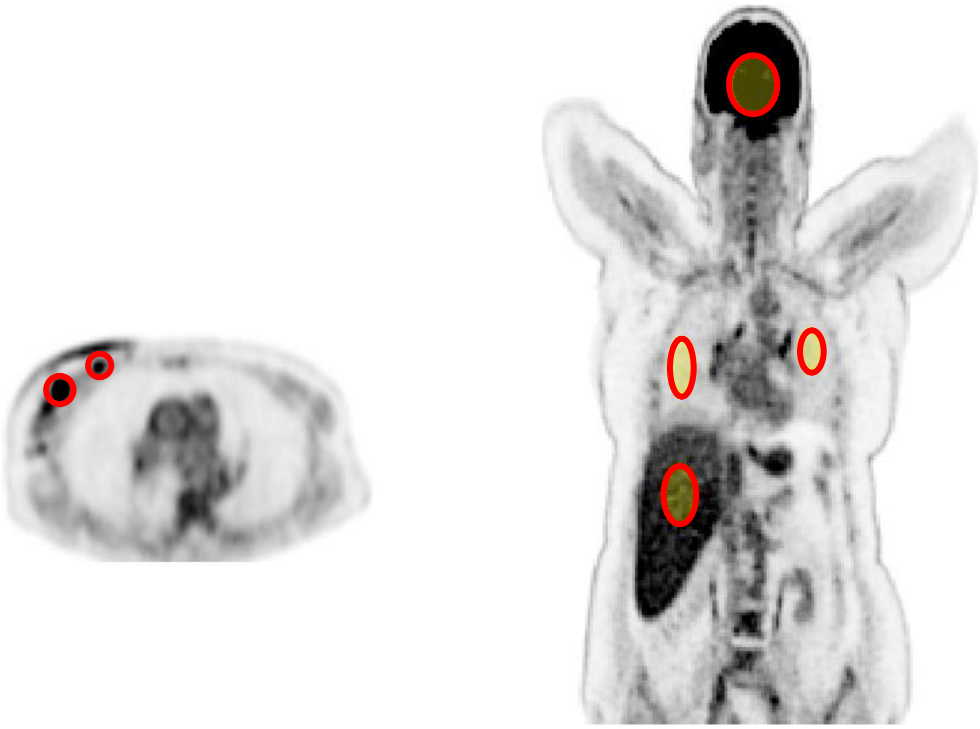

Supplemental figure 2. Representative ROIs drawn on malignant lesions (left) and the 4 ROIs defined on normal organs (right) including the brain, liver and right and left lungs.
